# Integrated Quasi-Optical Terahertz Liquid Sensor Leveraging Mode-Parity-Dependent Interaction with a Capillary-Confined Analyte

**DOI:** 10.3390/s25227026

**Published:** 2025-11-17

**Authors:** Andreas K. Klein, Julian Webber, Guillermo Carpintero, Masayuki Fujita, Daniel Headland

**Affiliations:** 1 Zentrum für Halbleitertechnik und Optoelektronik, Universität Duisburg-Essen, 47057 Duisburg, Germany; 2 Graduate School of Engineering Science, The University of Osaka, Osaka 560-8531, Japan; jwebber3@gmail.com (J.W.);; 3 Grupo de Optoelectrónica y Tecnología Laser, Universidad Carlos III de Madrid, 28911 Madrid, Spain; guiller@ing.uc3m.es; 4 Terahertz Engineering Laboratory, The University of Adelaide, Adelaide, SA 5005, Australia

**Keywords:** on-chip liquid sensor, terahertz and mm-waves, integrated terahertz optics

## Abstract

The integration of terahertz (THz) sensing technology into compact, on-chip platforms is essential to the advancement of high-precision chemical and biomedical analysis, promising to bring analytics closer to the point of care and to enable in situ analysis of industrial processes. This study presents an integrated quasi-optical THz liquid sensor that features a longitudinal cavity in a silicon slab waveguide, in which a capillary-confined analyte interacts with guided slab modes on resonance. The sensor design leverages mode-parity-dependent field distributions: even-parity resonances exhibit strong analyte-field interaction, whilst odd-parity modes remain largely unaffected by the presence of the analyte, enabling intrinsic self-calibration. The device is fabricated using deep reactive ion etching of high-resistivity silicon and monolithically integrates all required components. Experimental measurements with water and isopropanol demonstrate alternating resonance peaks with distinct sensitivity to refractive index and absorption, validated by linear shifts in frequency and transmission loss. The self-calibrating feature allows for real-time compensation of system fluctuations towards automated continuous monitoring applications. These findings establish the sensor’s capability for simultaneous, precise material characterization and calibration, highlighting its potential for in-line process monitoring and other high-bandwidth sensing applications.

## 1. Introduction

In recent years, the growing demand for high-precision sensing in fields such as chemical analysis, biomedical diagnostics, and environmental monitoring has spurred an interest in new sensor designs. Terahertz (THz) radiation, with its unique ability to sense intramolecular vibrations, is ideal to detect and distinguish numerous chemical compounds of interest [1,2,3,4,5]. However, the high in-medium attenuation of terahertz waves, especially in aqueous solutions, renders the acquisition of meaningful measured signals challenging [6,7,8]. Furthermore, for any given application, it will be necessary to move away from optical-bench setups towards integrated sensor solutions in order to allow for deployment in realistic application scenarios. For this reason, compact terahertz sensing platforms, and especially waveguide-based approaches, have received increasing interest in recent years [9,10,11,12]. A common route to achieve strong matter-field interaction is to eschew standard thin-film liquid measurements in favor of resonant structures, where the introduction of the analyte causes a frequency shift in the resonance and changes in Q-factor, yielding a large amount of information about the analyte [13,14,15]. However, one drawback of this approach is that spectroscopic information is lost, as a continuous broad bandwidth is generally required to identify spectral fingerprints. Furthermore, the need for resonance adds device complexity, as auxiliary components such as waveguides are commonly required to feed the integrated resonator. Each component comes with its own limitations upon operation bandwidth, which is a clear drawback in comparison to conventional free-space measurements. That said, in recent years, platforms addressing these drawbacks have been developed, offering quasi-optic beam properties with the well-defined experimental environment of an on-chip platform [16,17]. A convenient and viable approach to introduce an integrated microfluidic channel to this platform without disturbing guided waves has thus far proven elusive [14].

Here, we present and experimentally validate a new concept that improves upon prior approaches to resonator-based sensing by employing a longitudinal quasi-optical cavity that is several guided wavelengths-long in an integrated silicon slab waveguide chip, enclosed by partially reflective barriers that define a resonant Fabry–Perot cavity [18]. The size of this cavity is such that, over the 40% measurable spectral range, multiple resonances of both even- and odd-parity can be observed. Although this does not provide a continuous spectrum, it does offer numerous spectral sampling points, each of which offers unique information. An on-chip integrated sensor of this sort demands that the resonant cavity in question be implemented in a specific guided-wave structure, and in this case, a dielectric slab waveguide serves for this purpose. Modal fields are therefore unconfined in the in-plane dimensions of the chip itself, and so the propagation direction of guided waves must be manipulated using quasi-optical techniques, as a slab-mode beam [17]. This makes it possible to introduce a narrow capillary channel bearing liquid analyte directly into the path of the slab-guided beam whilst avoiding undesired interaction between THz waves and the microfluidic feeding apparatus.

The choice of a dielectric slab waveguide as the foundational device platform for this sensor makes it highly convenient to define a narrow microfluidic channel directly in the center of the Fabry–Perot cavity. At this point, odd-parity modes exhibit a node, meaning that the field magnitude is effectively zero in the vicinity of the analyte. Thus, the field interaction with the analyte is minimal, leading to transmission that is near-indiscriminate of the material inside the cavity. On the other hand, all even-parity modes exhibit an antinode, or maximum, at the position of the analyte, leading to a strong matter-field interaction that is enhanced by the resonance effect. As a result, resonance peaks alternate between a negligible and a strong response to the liquid analyte. This avails us of the possibility to utilize the unaffected (odd-parity) peaks as references, and so the sensor can self-calibrate with a single measurement to account for moment-to-moment fluctuations in the system, such as variations in transmitter power or receiver sensitivity in response to temperature. This would be particularly useful for continuous measurement applications, e.g., in-line monitoring of chemical engineering processes, where the flow of liquid analyte cannot realistically be stopped at regular intervals to allow for recalibration of the THz-range integrated quality-assurance system. As such, the concept that we introduce addresses the main practical limitations that are currently facing THz liquid-sensing applications, with an on-chip platform that is amenable to self-calibration during continuous inline measurement.

## 2. Materials and Methods

**Figure 1:**
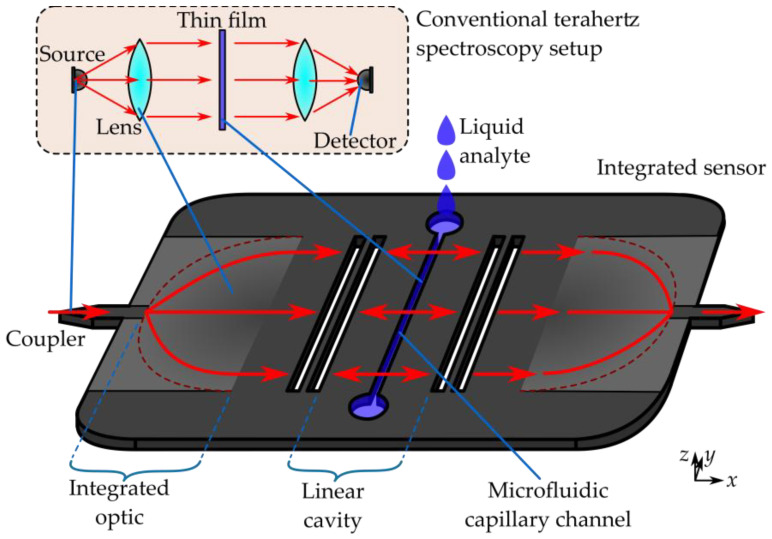
Illustration of the integrated sensor, in comparison to the corresponding components of a free-space setup (insert), with the corresponding components indicated. Radiation is injected via the coupler on the left. The beam is widened and collimated by the integrated optic, which acts as a lens. A Fabry-Perot cavity is formed by Bragg mirrors. In the center of the cavity is a microfluidic channel, which holds the liquid analyte. The beam is focused by the second integrated optic onto a coupler, where the radiation can be detected.

The integrated quasi-optical terahertz liquid sensor device that is the main subject of this work is fabricated from 200 μm-thick high-resistivity (>10 kΩcm) float zone silicon using deep reactive ion etching (DRIE). Manufacture was performed by Silicon Sensing Systems Ltd, Amagasaki, Hyogo, Japan. The photolithographic process used to create the mask for DRIE not only allows for precision well below 1% of the wavelength, but also wholly precludes the relative misalignment of any given feature as the entire structure is etched in parallel from a single mask layout. The core of the sensor system is a 20 μm-wide microfluidic channel that is etched all the way through the silicon slab. This channel runs across the center of a 2 mm-long cavity in the slab waveguide that is defined by Bragg mirrors at either side. The size of the cavity was chosen to have a minimum of three even and odd modes within the spectral range, to proof the functionality in more than a single spectral feature and to mitigate the risk of confounding a given spectral feature of interest with other spectral features, as discussed below for the even modes Each Bragg reflector is composed of a pair of rectangular ~227 μm-wide air-gaps, spaced ~75 μm apart, both of which being a quarter-wave in their respective media of interest, which interrupt the silicon slab and generate the partial reflections that make resonance possible. The slab-mode beam itself is launched and received by a pair of gradient index half-Maxwell fisheye lenses of 8 mm diameter, with the details of the specific design reported in [17]. These integrated quasi-optics are fed by a dielectric slot waveguide in order to effect a broadband transition from a dielectric channel waveguide to a point-source at the edge of the circumference. The off-chip connection is effected by a conventional tapered-spike coupler that performs progressive matching [16], which in this case is 2.8 mm-long. In this way, a single piece of intrinsic silicon monolithically integrates couplers, collimators, a cavity, and a liquid sensor—a complete terahertz system, as seen in Figure 1.

**Figure 2:**
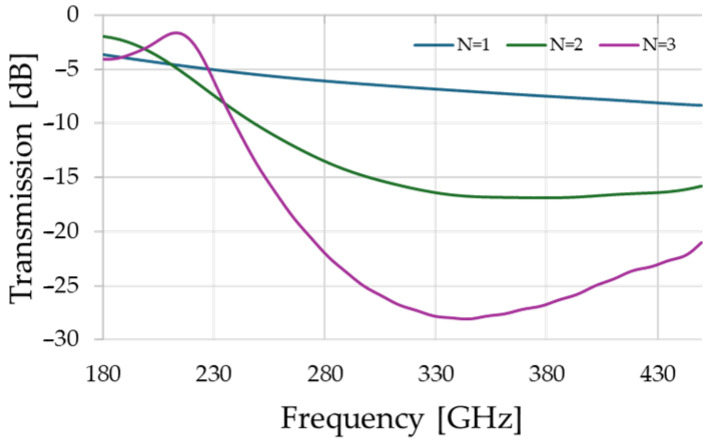
Transmission spectrum of a single Bragg-mirror for different numbers of air-gaps, *N*. These results are computed using the commercially available numerical full-wave electromagnetics simulation package, CST Studio Suite.

There is a tradeoff between resonance’s quality factor, which tends to increase with the number of air-gaps *N* in each Bragg mirror, and transmission efficiency, which is reduced by the very same parameter. This is because a greater number of air gaps will tend to increase the reflections and more effectively trap energy in the cavity, but at the same time, less energy is admitted into the cavity in the first place. In order to explore this tradeoff, the transmission of a single Bragg mirror is simulated using the commercial software package CST Studio Suite from Dassault Systèmes, Paris, France, and is presented in Figure 2. In the case of a single airgap, the transmission is too great to allow for adequate energy confinement when deployed in an optical cavity. On the other hand, a three-layer Bragg mirror exhibits transmission below 0.3%, which will ultimately compromise the signal-to-noise ratio in practice. Furthermore, the larger number of air gaps leads to increased frequency selectivity in the periodic structure, counter-intuitively yielding higher transmission than a single barrier at the lower-frequency end. In order to mediate between these extremes and produce an optimal design for our proof-of-concept sensor, we select *N* = 2. This results in a Bragg reflector with ~220 GHz bandwidth, and 3% transmission.

The samples were measured with an Anritsu VectorStar MS4647B (Atsugi, Japan) VectorNetwork Analyzer (VNA) equipped with Virginia Diodes frequency range extenders, which up-convert the VNA’s microwave-range signals to the THz range via electronic frequency multiplication. This instrumentation system targets the WR-3.4 band, which spans from 220 GHz to 330 GHz and has a dynamic range of up to 100 dB. As analytes, simple deionized water and isopropanol are used, with optical properties given in Table 1. The optical properties are the real and imaginary parts of the refractive index *n’* and *n*”, the absorption coefficient *α***,** and the absolute value of the refractive index |*n*|. While the attenuation of a resonant peak is influenced by *α* or, respectively, *n*”, the frequency shift is proportional to |*n*|.

**Figure 3:**
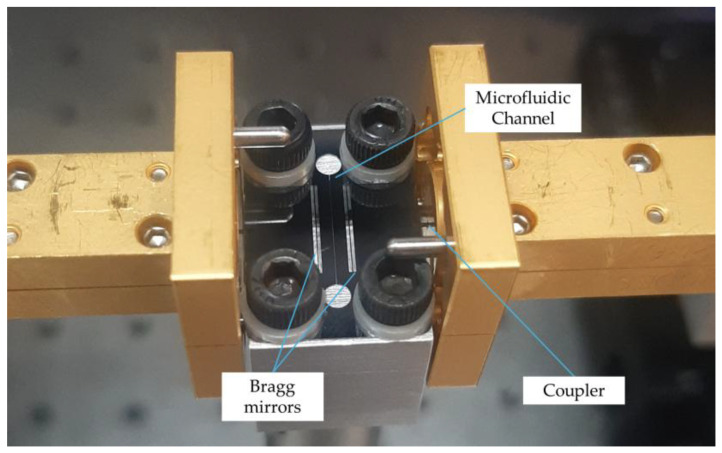
Photo of the quasi-optical liquid sensor during measurements. The sensor is connected to the WR-3.4 waveguides left and right for the transmission measurement, while being held on an aluminum holder with screws. The holder only touches the outer edges, so that the silicon is suspended for all parts where the electromagnetic field passes through. The liquid is added with a pipette via one of the circular apertures, where the capillary force draws the liquid into the channel and fills it completely.

The integrated sensor device is held in place with 4 screws, with one in each corner, atop a metal holder structure, as seen in Figure 3. The silicon rod transitions that terminate the integrated channel waveguides are inserted into the rectangular waveguides of our electronic instrumentation system, and precise micro-scale positioning is performed by affixing the THz-range frequency extension modules to micrometer-actuated translation stages and performing manual alignment guided by the naked eye. The liquid analyte is introduced by placing a drop with a handheld pipette into one of the circular wells, where capillary forces draw the liquid into the channel until the other aperture is reached. Due to the small amount of liquid, the liquid evaporates within ~2 min for the alcohol, and ~7 min for the water, leaving behind a bare sensor for which the measurement trace signal returns to that of the prior reference. As the time to complete the evaluation is considerably longer than the measurement time of around 5 s, we assume the measurements to be static, as no change in signal could be observed during consecutive back-to-back measurements until the point at which the analyte evaporates.

## 3. Results

The concept of the sensor centers around two key features of interest: The self-calibration via the full transmission of odd-parity modes, irrespective of the presence of an analyte, and the high sensitivity of the resonant cavity to the presence of the analyte for even-parity modes. Subsequently, we will present the results for these two features in two subsections.

### 3.1. Self-Calibration via Full Transmission for Odd Modes

**Figure 4:**
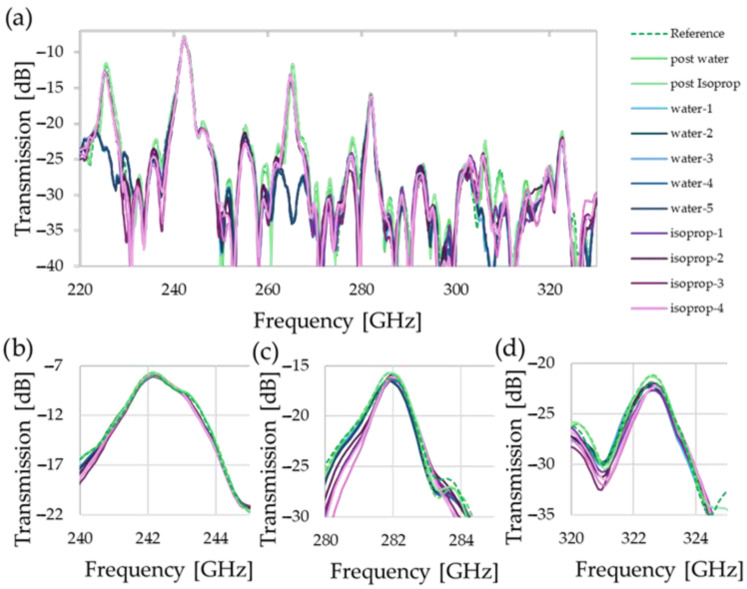
Frequency spectra of measurements with and without analyte present. Three empty measurements (green) and 5 measurements with water (blue) and isopropanol (purple) are shown. The full spectrum of the frequency extenders is shown in (**a**). The odd modes are shown in (**b**–**d**), where we see little change, indiscriminate of whether an analyte is present or not with as little as ~0.2 dB change for (**b**) and a change in ~1.8 dB for (**d**), where the overall signal strength is already strongly deteriorated from −8 dB to −21 dB at peak transmission.

Figure 4 shows the measurements of the empty liquid sensor before and after the measurements of analytes, as well as the traces for 5 measurements with each of the analytes—water and isopropanol—present. Although large resonators have an expected complex mode structure, some features are clearly visible in the full spectrum; we see multiple peaks with clear prominence above −10 dB, particularly at the lower frequency end. Here, we can discern between the even modes, for which there is considerable change between the measurements (approx. 230, 270, and 310 GHz), and odd modes, where there is no clear difference whether the analyte is present or not (approx. 240, 280, and 320 GHz). The overall slope of the spectrum, with maximum transition falling from −8 dB to −21 dB, can be explained by the increased radiation loss due to the larger free-space distance and increased scattering at the Bragg mirror layers at increased frequencies.

**Figure 5:**
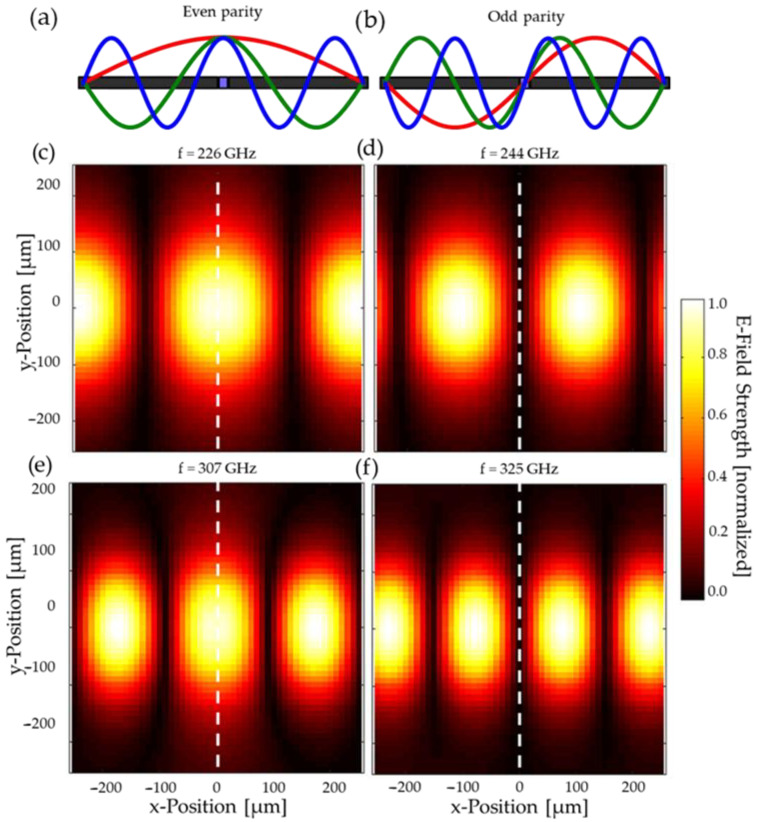
Dependence of channel interaction upon mode parity, showing (**a**,**b**) conceptual illustration of longitudinal cross-section, in which even-parity modes exhibit an antinode at the position of the analyte, whereas odd-parity modes exhibit a node, and (**c**–**f**) corresponding simulated electric fields at specific resonance frequencies. The even modes (**c**,**e**) show a clear maximum at the relevant positions, whilst odd modes (**d**,**f**) show a minimum. The position of the microfluidic channel, i.e., the center of the cavity, is indicated by the dashed line.

In order to provide insight into the mode-parity functionality of the resonator, Figure 5 shows the simulated electric field across the microfluidic channel for both odd and even modes. It can be seen that the center of the cavity is either overlapping with the center of a maximum (even-parity) in field strength, as in Figure 5c,e, or a minimum (odd-parity), as in Figure 5d,f. The size of these critical features of field morphology is significantly larger than the 20 µm of the channel, allowing for near-uniform field conditions across the volume of the liquid analyte. That says, as frequency is increased, the field pattern on-resonance becomes compressed, compromising this uniformity. Whilst this is beneficial for the sensing, as a larger share of the electric field overlaps with the microfluidic channel, hence increasing sensitivity, it is expected to become problematic for the self-calibrating feature at higher frequencies. As the calibration depends upon the negligible interaction of the analyte with the electric field at the minimum, this approximation is not true if the field-minimum’s lateral extension shrinks too far, as we already start to observe in Figure 5f.

### 3.2. Refractive Index Sensing via Even Mode Shift

**Figure 6:**
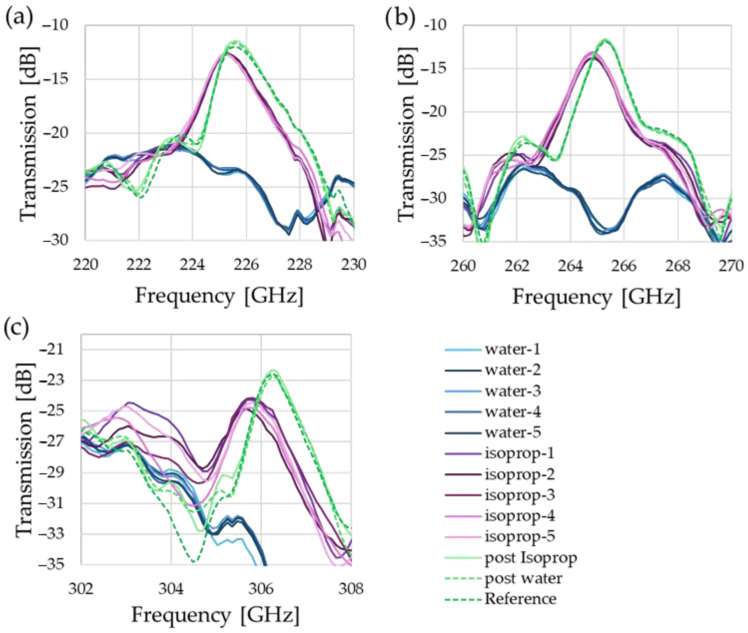
Comparison of the even modes. While (**a**,**b**) show a clear trend and easily identifiable peaks, the results for water in (**c**) coincide with another spectral feature, limiting the usability of this mode.

While self-calibration is realized via the odd modes, the even modes are leveraged to estimate the refractive index and losses. In the enlarged spectra around the peaks in Figure 6, all peaks shift to lower frequencies with increasing absolute value of the refractive index, as the optical path length, and hence the resonator’s electrical size, effectively increases. Simultaneously, attenuation is also observed, showing the clear difference between the relatively weak attenuation of isopropanol in comparison with the strongly absorbent water.

**Figure A1:**
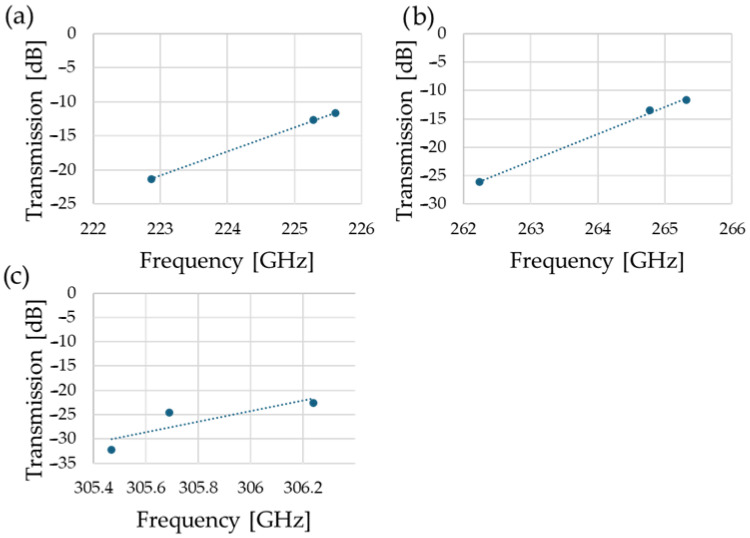
Plots of the transmission peaks for the different analytes at (**a**) resonance at ~225 GHz, (**b**) resonance at ~265 GHz, and (**c**) resonance at ~306 GHz. As an increasing refractive index leads to a decrease in frequency, the points correspond from left to right in each graph to water, isopropanol, and air. A linear regression (dashed line) was added to more easily identify deviations.

The evaluation of these results is shown in Table 2. Here we see that the sensitivity of the sensor varies for the different resonances. While the large sensitivity regarding |n| can be easily explained by the highest dynamic range. The three measurements at different frequencies also allow for comparison of trends, which is also useful to identify confounded data points—a common problem for sensors with complex mode structures. If a measurement peak coincides with a spectral feature, then the shift and attenuation will no longer be clearly discernible, resulting in a spectral range that cannot be used. One such case is expounded further in Appendix A, Figure A1.

The effect of the attenuation of the analyte, which is proportional to *α* or *n*”, can be clearly observed in the reduction in quality factor, while the shifts depend on the combination of real and imaginary components of the refractive index *n*, which both contribute to |*n*|. We observe that even the highly absorptive water still has a minimal Q-factor of 36, meaning aqueous solutions can be addressed as a target application for the sensor. The difference in quality factor changes between air, with an attenuation of *α* ≈ 0 cm^−1^, and water, with an *α* of 130 cm^−1^, ranges from 92 and 135 for the first two frequency ranges, following the trend that a larger quality factor results in increased sensitivity. The quality factor increases from 220 GHz to 300 GHz, due to the higher reflectivity of the Bragg reflectors in this range.

As for the accuracy of the measurements, the standard deviation across the whole spectrum for the measurements with an empty fluidic channel is 0.27 dB, for isopropanol it is 1.17 dB, and for water it is 0.41 dB. However, it is noted that this metric includes the region in-between resonance transmission peaks, and hence the impact of noise is greater, especially in the sharp dips. If we only examine the standard deviation around the peaks, e.g., 220 GHz–230 GHz for Figure 6a, the values are less than half.

## 4. Discussion

The results presented in the previous section show that simultaneous characterization of a liquid analyte and calibration of the system is indeed possible within the same measurement. While the present quasi-optical on-chip technology offers a very large bandwidth, the results also show that the dimensions of the microfluidic channel must be tailored to the desired frequency range to maintain the self-calibration properties. Overall, it can be stated that the full potential of the sensor platform will only be utilized with integrated large-bandwidth sources, although a full sweep may not be necessary if the source in question can target specific desired frequency samples in the vicinity of the resonances. It is noted that this discrete-frequency-sampling scenario would demand a high degree of thermal stability, which we see as likely, owing to the relatively short length of the cavity, combined with the low thermal expansion of silicon. Moreover, the full utilization of the broad bandwidth offered by the integrated quasi-optical slab-mode platform [17,21] would necessitate refinements to the resonator to enhance its broadband performance, i.e., for higher transmission and Q-factor. A larger bandwidth in instrumentation would also allow for a shorter quasi-optical cavity, which would yield larger frequency spacing between resonances. This would likely yield an increase in the overall sensitivity, in line with previously reported sensors that can achieve an order-of-magnitude larger shift in resonance frequency in terms of GHz/RIU [22,23]. On the other hand, a larger cavity, as in our present design, can cause adjacent resonances to overlap and confound each other somewhat. That said, it is beneficial that the self-calibrating nature of the sensor concept allows for reliable tracing of the absolute transmission, allowing us to continuously evaluate the attenuation of the peak without the need for reference measurements without the sample present. This technique would not be possible for prevailing conventional free-space terahertz sensing concepts. Further investigation is needed to see if the self-calibration feature can be maintained at the same quality when the quality factor of the cavity is increased. Currently, the main source of losses is radiation at the Bragg mirrors at increasing frequencies, which could potentially be addressed by utilizing a photonic bandgap medium in place of simple air gaps. That said, our present demonstration serves as an adequate proof-of-concept for self-calibrating on-chip terahertz capillary-confined liquid sensors.

## 5. Conclusions

We have presented a viable concept to realize compact terahertz on-chip liquid sensors, to address long-standing practical issues associated with bulky optical-benchtop terahertz systems. The frequency shift per refractive index unit (RUI) and the transmission loss per attenuation unit were both found to be linear and readily calculable from experimental data. These findings confirm the sensor’s high sensitivity to both refractive index and absorption changes, supporting its application in precise material characterization and sensing. Not only that, our sensor device also exploits mode parity-dependent interaction with a sample analyte in order to realize self-calibration, which will be of great benefit to inline monitoring, e.g., in a factory setting.

## Figures and Tables

**Table 1:** Table of analytes used, along with optical properties taken from [19,20], respectively.

|n|	n”	α	n’	Analyte
2.43	1.03	130	2.2	DI Water
1.73	0.17	21.5	1.72	Isopropanol

**Table 2:** Peak frequency and transmission averages for the three analytes, air (empty sensor), isopropanol, and water, and the calculated sensitivities for |n| and α, as shown in Appendix A.

Sensitivity α [dB/cm^−1^]	Sensitivity |n| [GHz/RUI]	Quality Factor	Maximum Transmission [dB]	Center Frequency [GHz]	Material
	−3.52	128	−11.63	225.61	Air
−0.076		121	−12.66	225.28	Isopropanol
		36	−21.35	222.86	Water
	−1.97	227	−11.71	265.32	Air
−0.113		185	−13.46	264.77	Isopropanol
		92	−26.15	262.24	Water
	−0.76 ^2^	309	−22.54	306.24	Air
−0.009 ^2^		213	−24.53	305.69	Isopropanol
		NA	−32.22 ^1^	305.47 ^1^	Water

^1^ Data potentially confounded by overlap with another spectral feature. ^2^ Confounded data excluded.

## Data Availability

Data is available from the authors upon reasonable request.

## Appendix A

### Linear Regression

As stated in Section 3, it is common for large resonators to have a complex spectral structure with a multitude of features that might overlap. This causes the data in the regions of overlap to be unreliable, which means these regions must be identified. In this case, our choice of optical cavity length is beneficial, as it offers three distinct odd/even mode pairs to allow for a comparison between them. This can help to identify potentially confounded data points, as the general trends are expected to be the same in all three plots, since the overall optical properties of the analytes do not drastically change in the measured frequency range. A simple approach for this is to plot the peaks, as shown in Figure A1. While there is a clear similarity between (a) and (b), the deviation in (c) can readily be identified, which is evidence of the aforementioned confounding effect.
